# Whole‐Genome Resequencing Reveals the Demographic History and Adaptive Evolution of *Tamarix austromongolica* in the Yellow River Basin

**DOI:** 10.1002/ece3.72666

**Published:** 2026-01-05

**Authors:** Shuai Gong, Jia Sun, Jianmin Chu, Hongxiao Yang, Honghao Gan, Qian Wang

**Affiliations:** ^1^ Research Institute of Forestry Chinese Academy of Forestry Beijing P.R. China; ^2^ Experimental Center of Desert Forestry Chinese Academy of Forestry P.R. China; ^3^ Qingdao Agriculture University Qingdao P.R. China

**Keywords:** adaptive evolution, demographic history, *Tamarix austromongolica*, whole‐genome resequencing, Yellow River Basin

## Abstract

*Tamarix austromongolica* is a native species widely used for ecological restoration in the Yellow River basin. Its natural distribution aligns with the river's strong environmental gradients. Previous research based on traditional molecular markers suggested that the Yellow River acts as an efficient corridor for gene flow, leading to weak genetic differentiation in the species. However, a recent genotyping‐by‐sequencing study indicates a more complex genetic structure. This discrepancy reveals the limitations of low‐resolution genetic markers in resolving genetic differentiation against a background of strong gene flow. Here, we utilized whole‐genome resequencing to analyze genomic variation data from 112 samples collected from 20 populations in the Yellow River Basin. Our results revealed three distinct genetic lineages, each corresponding to a specific habitat: the Liujiaxia lineage (high‐altitude), the Hetao lineage (arid), and the Sanmenxia lineage (saline‐alkali). Demographic modeling indicated that the Liujiaxia lineage diverged first, approximately 470,000 years ago, likely due to glaciation‐induced isolation. Furthermore, significant asymmetric gene flow was detected between the Hetao and Sanmenxia lineages, highlighting the role of the river as a corridor for genetic exchange. Genome‐wide scans identified candidate genes associated with local adaptation in each lineage, with functions primarily related to diverse environmental stress responses, DNA damage repair, and pathogen defense. These results provide a foundation and resources for understanding the mechanisms of river‐driven plant differentiation and the stress‐resistant breeding of *T. austromongolica*.

## Graphical abstract

Whole‐genome resequencing of *Tamarix austromongolica* in the Yellow River Basin reveals three distinct genetic lineages, each corresponding to a specific local environment (high‐altitude, arid, or saline‐alkali). The identification of candidate genes underlying these patterns provides a genomic foundation for understanding plant evolution in river systems and for developing stress‐resistant varieties.

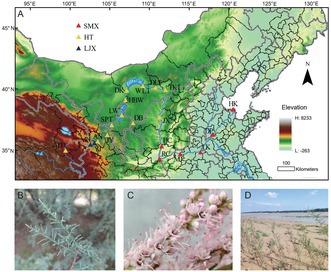

## 1 Introduction

The geomorphological evolution of major river systems is a key driver shaping the genetic diversity, population structure, and adaptive differentiation of riparian plants (Yue et al. 2012; Davis et al. 2018; Higgisson et al. 2020). The complex topography of river corridors creates dynamic patterns of population isolation and connectivity (Werth et al. 2014, Imbert and Lefèvre, 2003). These patterns, coupled with pronounced upstream‐downstream ecological gradients, impose a mosaic of selective pressures that profoundly influence a species' evolutionary trajectory (Mitsui and Setoguchi 2012). Understanding the genetic structure and adaptive mechanisms of plants in major river systems is essential for the utilization of germplasm resources and the practice of ecological restoration.

The Yellow River, a world‐class mega‐river, was formed approximately 1.25 million years ago through a series of channeling and rerouting events (Li et al. 2023). Originating on the Tibetan Plateau and flowing through the Loess Plateau before emptying into a coastal delta, its vast geographical span and altitudinal drop create extreme environmental gradients ranging from alpine cold to arid and saline conditions (Brierley and Li 2013; Brierley et al. 2016). This makes it an ideal setting to explore plant adaptive evolution under heterogeneous conditions. *Tamarix austromongolica* (Tamaricaceae), a native species widely used in ecological restoration across the Yellow River Basin, exhibits remarkable tolerance to drought, salinity, and nutrient‐poor soils (Yang et al. 2018; Wei et al. 2020). Its natural distribution is tightly coupled with the river's ecological gradients, and its reliance on wind and water for seed dispersal makes it an excellent model for elucidating plant responses to the complex selective pressures of riverine environments (Liu 2012). Previous research based on traditional molecular markers (e.g., simple sequence repeat and chloroplast DNA) has generally suggested that the Yellow River system acts as an efficient dispersal corridor, promoting extensive gene flow and resulting in weak overall genetic differentiation for the species (Liang et al. 2018; Wen et al. 2020). These studies also pointed to a significant population expansion originating from the upper reaches (Liang et al. 2018, Wen et al. 2020). However, a recent, higher‐resolution genotyping‐by‐sequencing study preliminarily revealed two genetic lineages, suggesting the presence of previously undetected barriers to gene flow (Gong et al. 2024). This discrepancy underscores the limitations of low‐resolution genetic markers in resolving population structure against a background of strong gene flow and in resolving demographic history against a complex geological and climatic background (Davey et al. 2011).

Building upon the insights from previous studies and considering the complex evolutionary history of the Yellow River, we hypothesized that (1) high‐resolution, whole‐genome data would reveal a distinct genetic structure corresponding to the sharp ecological gradients along the river, with these genetic lineages exhibiting signals of local adaptation to their specific environments, and that (2) the species' demographic history is more complex than a simple “upstream origin‐downstream expansion” model, with different genetic lineages possessing unique demographic histories. To test these hypotheses, we conducted whole‐genome resequencing of 112 *T. austromongolica* samples from 20 populations in the Yellow River Basin. Our specific objectives were to (a) resolve the population genetic structure, test its correspondence with ecological gradients, and identify potential signals of selection associated with specific environments; and (b) reconstruct the species' demographic history to explore the complex impacts of the Yellow River's geological evolution and paleoclimatic changes. This research provides new insights into the evolutionary mechanisms underlying divergence and adaptation in riverine ecosystems and offers valuable genetic resources and theoretical foundations for the restoration of degraded ecosystems and the breeding of stress‐tolerant plants.

## 2 Materials and Methods

### 2.1 Sampling and Whole‐Genome Resequencing

**FIGURE 1:**
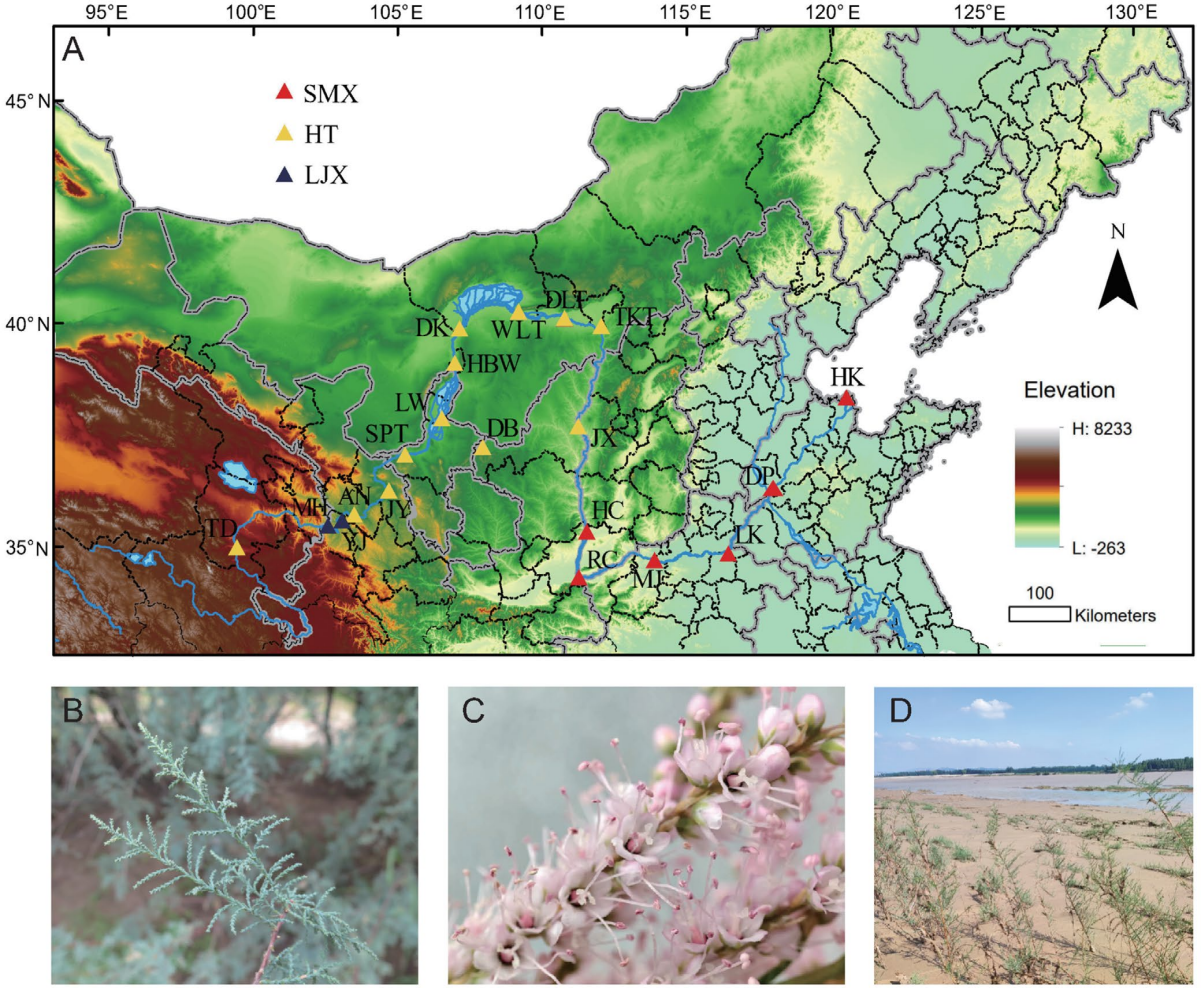
Distribution and morphology of *Tamarix austromongolica*. (A) Map showing the sampling locations. (B) Cauline and leaf. (C) Flower. (D) Natural habitat at the KTK site.

Natural populations of *T. austromongolica* were identified through consultations with local forestry authorities and villagers, combined with field investigations. During the flowering period (June–August), young leaves were collected from 112 individuals across 20 *T. austromongolica* populations in eight provinces traversed by the Yellow River for whole‐genome resequencing (Figure 1; Table S1). To avoid sampling clonal individuals, a minimum distance of 30 m was maintained between sampled individuals within a population, and different populations were separated by at least 15 km. Leaves were rapidly dried in silica gel, sealed in plastic bags, and the geographical location of each sampling site was recorded. Species identification was based on floral and foliar morphological characteristics. Representative germplasm resources from multiple habitats were deposited at the *T. austromongolica* Resource Garden of the Coastal Forestry Research Center of the National Forestry and Grassland Administration in Weifang, Shandong Province (37°0′15.96″ N, 119°7′4.41″ E). High‐quality genomic DNA was extracted from leaf tissues using a modified cetyltrimethylammonium bromide (CTAB) protocol. DNA concentration and purity were quantified using a NanoDrop 2000 spectrophotometer (NanoDrop Technologies, Wilmington, DE, USA) and a Qubit dsDNA HS Assay Kit on a Qubit 3.0 fluorometer (Life Technologies, Carlsbad, CA, USA). DNA integrity was assessed through electrophoresis on a 0.8% agarose gel. Sequencing libraries were subsequently constructed from genomic DNA using the VAHTS Universal DNA Library Prep Kit for MGI (Vazyme, Nanjing, China), and library quality and size distribution were confirmed using the Qubit 3.0 fluorometer and Bioanalyzer 2100 system (Agilent Technologies, CA, USA). Finally, libraries were sequenced on the MGISEQ 2000 platform (BGI, China) to produce paired‐end 150‐bp reads targeting a minimum genome coverage depth of 20×. DNA extraction, library preparation, and sequencing were conducted by Frasergen Bioinformatics Co. Ltd. (Wuhan, China). Publicly available whole‐genome sequencing data for *Tamarix chinensis* (GenBank Accession: GCA_030549775.1) were retrieved from the NCBI GenBank database for use as an outgroup in phylogenetic analyses (Sun et al. 2016).

### 2.2 SNP Calling

Whole‐genome short reads were trimmed with Trimmomatic v0.39 (Bolger et al. 2014) and aligned to the chromosome‐level *T. austromongolica* reference genome (GenBank Accession: GCA_039764185.1) using BWA‐MEM v0.7.17 (Li and Durbin 2009). The resulting alignments were converted to BAM format, sorted, and duplicates removed with SAMtools v1.16 (Li et al. 2009). Variant calling was performed using GATK v4.1.4.0 (DePristo et al. 2011) to generate gVCF files containing variant sites and associated sequencing depths. We combined samples using the CombineGVCFs module, and subsequently produced VCF files with GenotypeGVCFs. Variants were filtered through multiple steps: (i) GATK VariantFiltration tool was used to remove false positives by setting thresholds for quality by depth (QD < 2.0), Fisher strand bias (FS > 60.0), and mapping quality (MQ < 40.0), and specifying parameters for variant clustering (−‐cluster‐window‐size 5, −‐cluster‐size 2). (ii) SNPs within 5 bp of gaps and within a 10 bp window surrounding contiguous gaps were excluded using the vcfutils.pl varFilter script. (iii) Further quality control was performed with VCFtools v0.1.13 (Danecek et al. 2011), applying thresholds for minimum depth (‐‐minDP 4), maximum depth (‐‐maxDP 1000), minimum quality (‐‐minQ 30), minimum genotype quality (‐‐minGQ 80), minor allele frequency (‐‐maf 0.05), maximum allele count (‐‐max‐alleles 2), and maximum missing rate (‐‐max‐missing 0.7). The resulting high‐quality SNPs were annotated using ANNOVAR v3.5 (Wang et al. 2010) with the *T. austromongolica* genome annotation file. The resulting high‐quality SNPs were used as a general dataset for several analyses. For specific analyses with distinct model assumptions, further filtering was applied (Table S2).

### 2.3 Population Structure and Differentiation

Quality control was performed on the variant call format (VCF) file using PLINK v1.90 (Purcell et al. 2007), removing single‐nucleotide polymorphisms (SNPs) with a missing data rate > 10% and a minor allele frequency (MAF) < 5%. The filtered dataset was then pruned for linkage disequilibrium (LD) with an *r* ^2^ threshold of 0.2, using a window of 50 SNPs and a step size of 10 SNPs. Principal component analysis (PCA) was performed in PLINK to visualize genetic structure (Patterson et al. 2006), and the optimal number of clusters was determined by a *K*‐means analysis. The optimal *K* was identified by calculating the maximum distance to a line connecting *K* = 1 and *K* = 10 on the Within‐Cluster Sum of Squares (WCSS) curve. We further examined population structure using ADMIXTURE v1.3.0 (Alexander et al. 2009), testing ancestral population numbers from *K* = 1 to 10 with five replicates per *K* value, and determined the optimal *K* based on the lowest cross‐validation error. Phylogenetic relationships were reconstructed by building a maximum likelihood (Felsenstein 1981) tree in IQ‐TREE2 v2.2.1 (Minh et al. 2020) with the GTR model, 1000 bootstrap replicates, and BNNI algorithm for topology optimization, and the resulting tree was visualized using iToL v5 (Letunic and Bork 2021). Finally, nucleotide diversity (*π*) and fixation index (*F*_ST_) between populations were calculated with VCFtools using a 100 kb window size and 10 kb step size to quantify genetic diversity and differentiation.

### 2.4 Demographic Modeling

To investigate the demographic history of *T. austromongolica*, we first performed Pairwise Sequentially Markovian Coalescent (PSMC) (Li and Durbin 2011) analysis on high‐quality individuals with sequencing depth > 30× using default parameters, with 100 bootstrap replicates per individual. For these analyses, we applied a generation time of 3 years and a mutation rate of 6.46 × 10^−9^ substitutions/site/year, which was estimated based on the synonymous substitution rate (Ks) of homologous blocks between *Vitis vinifera* and *T. austromongolica* (*μ* = Ks/2 T), rather than the general mutation rate for the Tamaricaceae family (7.62 × 10^−9^ /site/year). Recent demographic dynamics were further inferred using the Sequentially Markovian Coalescent model (SMC++) (Terhorst et al. 2017). After excluding outlier individuals (AN2, AN3, LW1, and MH6), we masked genomic regions with missing data and converted variant data from VCF format into SMC++ input with the vcf2smc tool, again using the same values for generation time and mutation rate as described above. Finally, we modeled the population history using dadi (Gutenkunst et al. 2009) based on the three‐dimensional joint site frequency spectrum (3D‐JSFS). To construct the 3D‐JSFS, autosomal SNPs were filtered to retain loci present in 90% of individuals, remove linked sites, and were thinned to one SNP per 10 kb window using VCFtools (‐‐thin 10,000) to ensure site independence (Excoffier et al. 2013; Choi et al. 2020). We tested 64 demographic models (Barratt et al. 2018, Firneno Jr et al. 2020), comprising 32 for each of two competing topologies: one with the Liujiaxia lineage as the basal split, and the other with the Hetao lineage. The best‐fitting model was selected based on the lowest Akaike Information Criterion (AIC) value, with model parameters scaled to absolute values using the aforementioned generation time and mutation rate.

### 2.5 Genome‐Wide Selection Scan

To identify genomic regions under selection, we calculated the pairwise *F*_ST_ between populations and the *π* within each population using VCFtools. Calculations were performed with a sliding window of 100 kb and a step size of 10 kb. Additionally, cross‐population composite likelihood ratio (XP‐CLR) analyses were conducted for each chromosome using the xpclr package v1.1.2 (Chen et al. 2010). Candidate genomic regions were defined as windows that simultaneously fell within the top 5% of *F*_ST_ and XP‐CLR scores and the bottom 5% of the *π* values. Genes located within these candidate regions were then functionally annotated using eggNOG‐mapper v5.0 to investigate their potential roles in the local adaptation of *T. austromongolica* (Huerta‐Cepas et al. 2019).

## 3 Results

### 3.1 Sequencing, Mapping, and SNPs Calling Statistics

Whole‐genome sequencing generated an average of 311,306,559 raw reads per individual, with 287,207,741 clean reads retained on average after quality filtering. These clean reads were aligned to the *T. austromongolica* reference genome, resulting in an average mapping rate of 99.14% (range: 94.37%–99.67%) and an average sequencing depth of 31.59× (range: 23.39×–48.67×) across all samples (Table S1). Following rigorous SNP filtering and annotation using ANNOVAR, we identified 4,652,019 high‐quality SNPs distributed across 12 chromosomes. The highest proportion of these were located in intergenic regions (3,575,178; 76.85%), followed by those in intronic (642,330; 13.81%) and exonic (119,314; 2.56%) regions (Figure S1; Table S3).

### 3.2 Genetic Diversity and Population Structure

**FIGURE 2:**
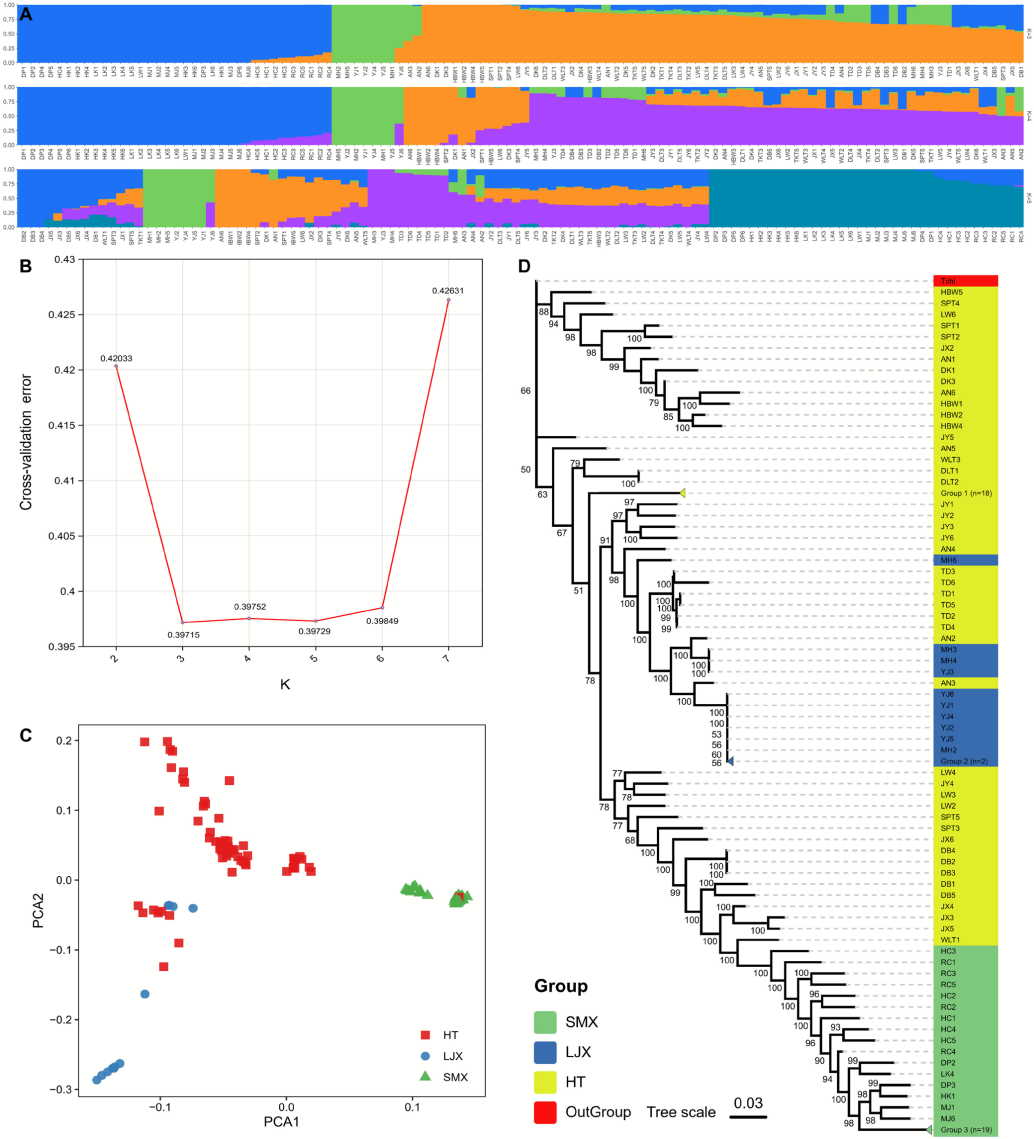
Genetic structure and phylogenetic relationships of *Tamarix austromongolica*. (A) Population structure of *T. austromongolica* individuals based on ADMIXTURE analysis with *K* values of 3–5. (B) Cross‐validation error at different *K* values in ADMIXTURE. (C) Principal component analysis plots of the first two principal components for the 112 samples of *T. austromongolica*. (D) Maximum likelihood phylogenetic tree of *T. austromongolica* with *Tamarix chinensis* as the outgroup. Clades with bootstrap values less than 50 were collapsed. Group 1 (*n* = 18) consists of accessions DK2, DK4–6, DLT3–5, HBW3, JX1, LW5, TKT1–5, WLT2 and WLT4–5. Group 2 (*n* = 2) consists of MH1 and MH5. Group 3 (*n* = 19) consists of DP1, DP4–6, HK2–6, LK1–3, LK5–6, LW1 and MJ2–5.

The ADMIXTURE analysis revealed an optimal genetic clustering of three groups (*K* = 3) for the *T. austromongolica* populations in the Yellow River Basin, as indicated by the lowest cross‐validation error. These three lineages were designated as Liujiaxia (LJX), Hetao (HT), and Sanmenxia (SMX). Although models with *K* = 4 and *K* = 5 suggested the separation of several populations (HBW, SPT, DB, and JX) from the HT lineage (Figure 2A,B), these subdivisions were not supported by pairwise differentiation analysis. This analysis revealed no significant genetic divergence from the main HT group (*F*_ST_ < 0.05; Figure S2). Principal component analysis further supported the three‐lineage model, and *K*‐means clustering analysis indicated that *K* = 3 was the optimal number of clusters (Figure S3 and Figure 2C). Genetic diversity analysis revealed the highest nucleotide diversity in the HT lineage (*π* = 7.3 × 10^−4^), followed by the SMX (*π* = 5.1 × 10^−4^) and LJX (*π* = 4.7 × 10^−4^) lineages. Estimates of genetic differentiation indicated the greatest differentiation between the LJX and SMX lineages (*F*_ST_ = 0.29), while each exhibited moderate differentiation from the HT lineage (*F*_ST_ = 0.10 and 0.13, respectively; Figure S4). Based on comprehensive evaluations of population structure, geographic distribution, and pairwise differentiation analysis, we classified *T. austromongolica* in the Yellow River Basin into three genetic lineages: LJX, HT, and SMX. Subsequently, to investigate the divergence history of these three lineages (Figure 2D), we constructed a Maximum Likelihood phylogenetic tree. The results indicate that while LJX and SMX each formed monophyletic lineages with high bootstrap support (Bootstrap Support = 100), the HT lineage exhibited paraphyly, and the deep nodes connecting the major lineages had low support. We hypothesize that strong gene flow between populations interfered with the ML tree algorithm's inference of ancestral origins, thereby preventing the HT lineage from resolving a clear phylogenetic topology. This gene flow hypothesis is further supported by our subsequent dadi analysis results (Section 3.3). Moreover, phylogenetic analysis and principal component analysis both consistently identified the same outlying individuals (AN2, AN3, LW1, and MH6) that were sampled from key nodes representing the geomorphological evolution of the Yellow River (Figure 2C,D).

### 3.3 Demographic History Inference

**FIGURE 3:**
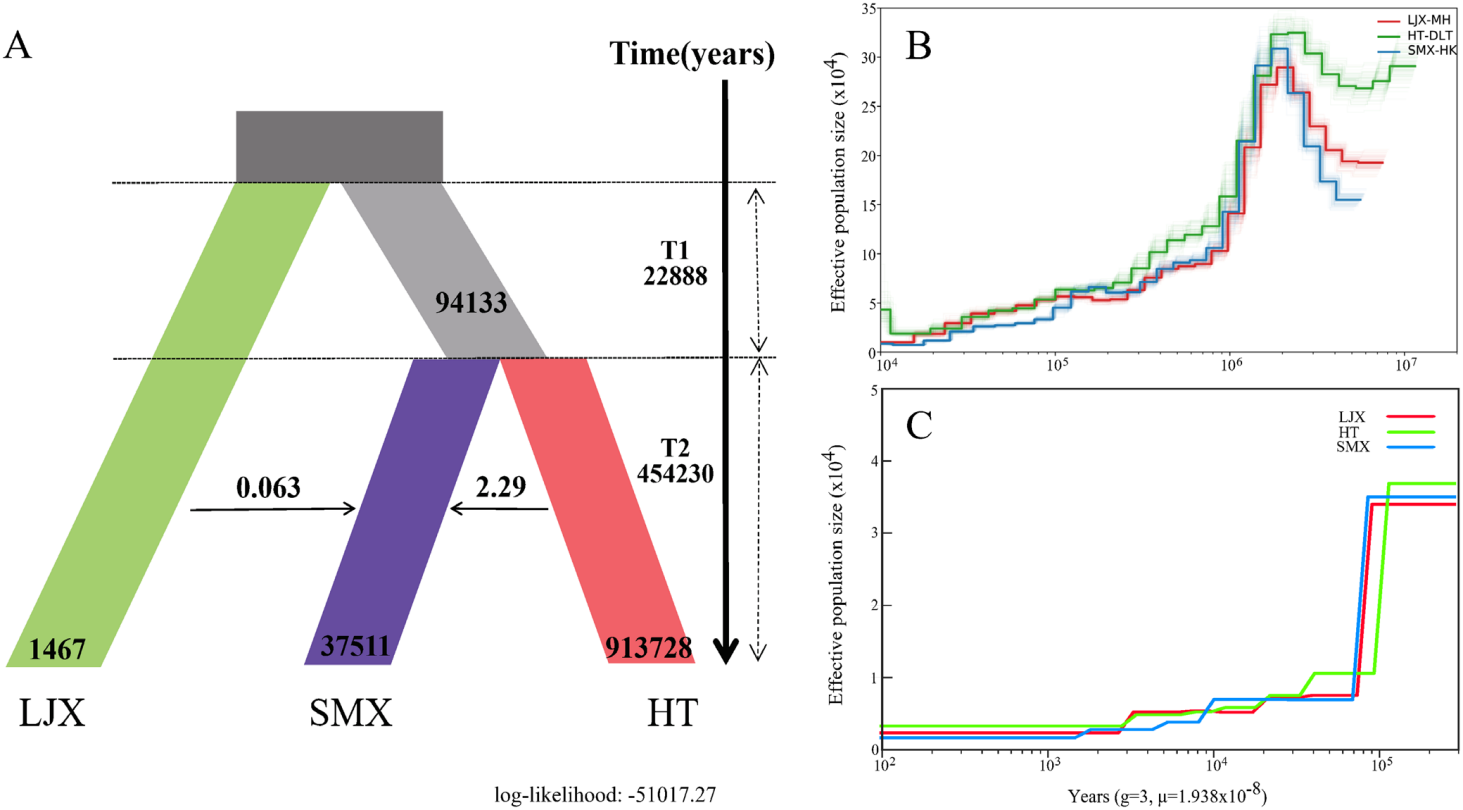
Demographic history of *Tamarix austromongolica* lineages. (A) Schematic diagram of the optimal model for the phylogenetic diversification of *Tamarix austromongolica* simulated with dadi. (B) PSMC analysis of changes in effective population size among different genetic groups of *T. austromongolica*. (C) SMC++ analysis of changes in effective population size among different genetic groups of *T. austromongolica*.

The software dadi was used to evaluate and compare multiple predefined demographic models to investigate the origin and divergence history of *T. austromongolica*. The refugia_adj_2_var_uni model, which posits the LJX lineage as the earliest to diverge, was identified as the best‐fitting model according to its highest log‐likelihood and lowest AIC values (Table S4). Parameters from the optimal model indicate that the LJX lineage split from the ancestral population approximately 470,000 years ago and subsequently maintained a relatively small effective population size. Subsequently, the SMX and HT lineages diverged about 450,000 years ago, after which the HT lineage underwent a pronounced expansion, whereas the SMX lineage retained a moderate population size. The model also revealed significant asymmetric gene flow, with a high migration rate from SMX to HT but limited gene flow from LJX to SMX (Figure 3A). Furthermore, we employed the PSMC analysis to track historical trends in effective population sizes, which revealed an overall declining trend since approximately 1 million years ago. Similarly, recent population size estimates obtained using SMC++ did not detect the expansion of the HT lineage (Figure 3B,C). This apparent discrepancy between dadi and PSMC/SMC++ results may be attributable to the strong gene flow detected in the dadi model, as pervasive introgression can obscure or mask population expansion signals in coalescent‐based analyses such as PSMC and SMC++ (Hawks 2017; Novo et al. 2023).

### 3.4 Investigating Potentially Adaptive Divergence

**FIGURE 4:**
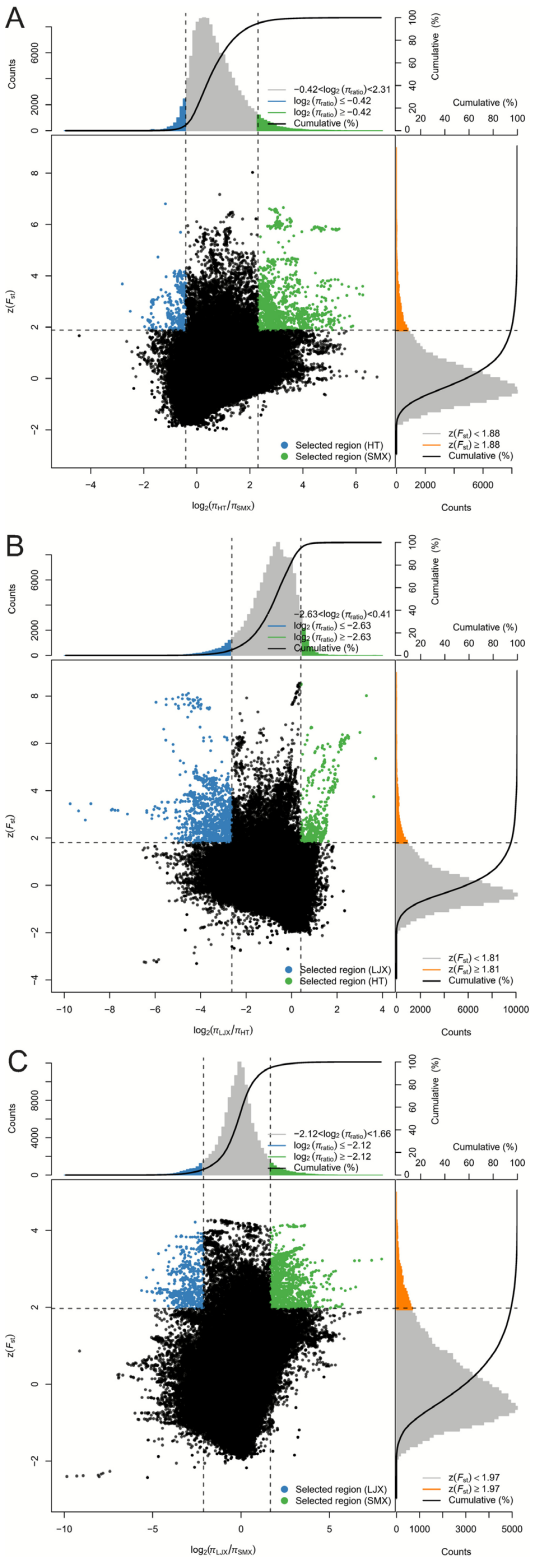
Distribution of log2π Ratios and *F*_ST_ Values among *Tamarix austromongolica* lineages. (A) HT versus SMX. (B) LJX versus HT. (C) LJX versus SMX.

**FIGURE 5:**
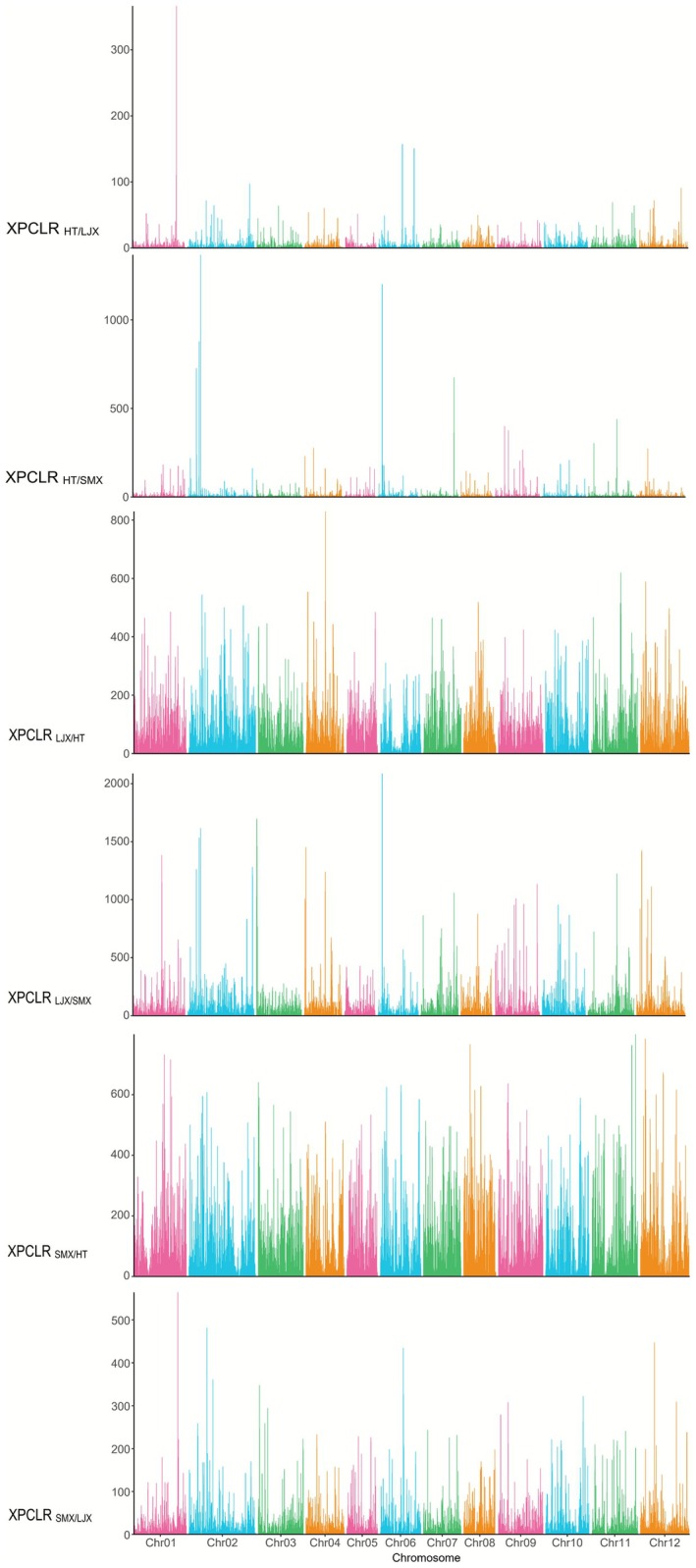
Distribution of XPCLR Values among Tamarix austromongolica lineages. (A) HT versus LJX. (B) HT versus SMX. (C) LJX versus HT. (D) LJX versus SMX. (E) SMX versus HT. (F) SMX versus LJX.

Genome‐wide scans revealed multiple loci under selection associated with local adaptation among different populations (Figures 4 and 5). We identified 392, 205, and 545 candidate genes under selection in the LJX, HT, and SMX lineages, respectively (Tables S5–S7). In the high‐altitude LJX lineage from the eastern Qinghai‐Tibet Plateau, candidate genes were significantly enriched in GO terms related to cellular response to decreased oxygen levels (GO:1901575) and light stimulus (GO:0071482) (Table S8). Furthermore, the light‐stress–associated gene *TOCC* (*evm.TU.Chr02.2121*) was also identified in this lineage (Table S5). For the HT lineage, inhabiting the arid and semi‐arid regions of the Inner Mongolian Plateau, candidate genes were enriched for pathways such as response to stimulus (GO:0050896) and DNA synthesis involved in DNA repair (GO:0000731), and the drought‐stress–related gene *RDUF2* (*evm.TU.Chr04.1620*) was detected (Tables S6 and S9). In the SMX lineage, which is predominantly distributed in the saline‐alkali area of the Yellow River Delta, candidate genes were predominantly enriched in the regulation of defense response to bacterium/fungus (GO:1900424/GO:1900150) and stress response to metal ion (GO:0097501) (Table S10). The salt‐stress–related gene *TSN1* (*evm.TU.Chr11.868*) was identified (Table S7).

## 4 Discussion

### 4.1 Adaptive Divergence in *T. austromongolica* Along Ecological Gradients

This study found that the three identified genetic lineages of *T. austromongolica* from the Yellow River Basin correspond closely to distinct ecological gradients. The LJX, HT, and SMX lineages inhabit the high‐altitude river source region, arid and semi‐arid inland areas, and saline‐alkali lands, respectively. These distinct ecoregions impose markedly different selective pressures, encompassing climatic factors (temperature, light), soil conditions (nutrient limitation, salinity), and hydrological features (drought, flooding). Genome‐wide selection scans have revealed that the LJX lineage is enriched for genes involved in cellular responses to hypoxia and light stimulus, consistent with adaptation to the high‐altitude, low‐oxygen, and high‐radiation environment. The candidate gene *TOCC* (*evm.TU.Chr02.2121*), involved in the synthesis of antioxidants for photoprotection (Porfirova et al. 2002), further supports this adaptive mechanism. In the HT lineage, selection signatures were enriched in pathways associated with drought stress response and DNA repair. The candidate gene *RDUF2* (*evm.TU.Chr04.1620*), a positive regulator in ABA‐mediated drought response, likely enhances drought resistance by augmenting the ABA signaling pathway (Kim et al. 2012). Concurrently, the enrichment of DNA repair pathways suggests an evolved capacity to mitigate drought‐induced DNA damage. The SMX lineage exhibited selection in pathways associated with salt and biotic stress resilience. The candidate gene *TSN1* (*evm.TU.Chr11.868*) was found to facilitate growth under saline conditions by stabilizing gibberellin synthase mRNA (Yan et al. 2014). The enrichment of pathways associated with biotic defense and metal ion response suggests a multifaceted adaptation to both primary salt toxicity and associated secondary stresses, such as increased susceptibility to pathogens. In summary, the present findings systematically elucidate the mechanisms of adaptive divergence in *T. austromongolica* along environmental gradients. These results provide valuable genetic resources and theoretical insights for the restoration of degraded ecosystems and the breeding of stress‐resistant plants.

### 4.2 The Dual Role of the Yellow River in Shaping the Phylogeography of *T. austromongolica*

By integrating demographic history simulations, phylogenetic relationships, and geospatial distribution, this study systematically reveals the dual role of the Yellow River as both a barrier and a corridor in shaping the phylogeographic pattern of *T. austromongolica*. Demographic modeling with dadi inferred that the divergence of the upstream LJX lineage occurred approximately 477,000 years ago (Figure 3A), which corresponds with the Middle Pleistocene MIS12 glacial period (Zhou et al. 2002, 2006). The distribution of LJX lineage in the Qilian Mountains, which served as a center of MIS12 glaciation (Zhou et al. 2011), suggests that intense glacial activity likely drove ancestral populations into refugia, causing allopatric differentiation. This conclusion is further supported by the persistence of ancient and unique chloroplast haplotypes within this lineage (Liang et al. 2018; Wen et al. 2020). In contrast, the middle and lower reaches of the Yellow River acted as a corridor for gene flow. Dadi models indicated significant genetic exchange between the HT and SMX lineages (Figure 3A), a process likely facilitated by the region's gentler topography. This genetic connectivity is corroborated by the phylogenetic placement of several individuals that are geographically distant from their primary lineage (Figure 2B,C). However, despite its position within this corridor, the downstream SMX lineage exhibited the lowest genetic diversity. This pattern is probably attributable to historical founder effects and ecological disturbances. First, a signal of rapid population expansion supports the hypothesis that pioneer ancestors underwent an initial genetic bottleneck during their eastward migration (Liang et al. 2018; Letunic and Bork 2021). Second, recurrent channel avulsions in the lower Yellow River repeatedly reset local populations (Li et al. 2024; Zheng et al. 2017). This process hindered the effective accumulation of alleles from upstream, thus maintaining the genetic impoverishment established by the founder effect. In summary, the phylogeographic pattern of *T. austromongolica* has been shaped by the combined effects of historical glacial isolation, habitat heterogeneity, and the dual role of the river as both a barrier and a corridor. These findings provide an important case study for understanding the complex mechanisms through which major river systems drive species differentiation.

## 5 Conclusion and Future Perspectives

In this study, we employed whole‐genome resequencing to elucidate the evolutionary history and adaptive divergence of *T. austromongolica* within the complex environmental gradients of the Yellow River basin. Our results revealed three distinct genetic lineages, whose distributions correspond to three major ecological regions: high‐altitude, inland arid, and saline‐alkali coastal, respectively, and exhibit signals of local adaptation to these specific environmental stresses. Furthermore, our demographic history reconstruction clarified that the species' complex evolutionary trajectory was not a simple “upstream origin‐downstream expansion.” The early allopatric differentiation of the LJX lineage may have originated from geographic barriers formed in the upper Yellow River during Middle Pleistocene glacial periods, while the middle and lower river channels acted as a corridor promoting gene flow between the HT and SMX lineages. Finally, we attribute the lower genetic diversity of the downstream SMX lineage to the combined effects of historical founder effects and ecological disturbances caused by frequent channel avulsions.

The results of this study should be interpreted within the context of its limitations, which also provide new perspectives for future research. First, the sample size per geographical population (average 5–6 individuals) constrained the precise estimation of intrasite genetic diversity parameters. Increasing sampling density per plot in future studies will facilitate more refined analysis of population dynamics at the plot scale. Second, regarding the history of the LJX lineage, future supplementation with samples from the western Qilian Mountains population would enable more precise verification of historical dynamics within this lineage and provide a more complete picture of its long‐term isolation. Finally, the functions of the candidate genes identified in this study require further validation through functional experiments to provide a scientific basis for integrated stress‐resistant breeding and adaptive provenance selection in T. austromongolice, bolstering the resilience of ecosystem restoration (Breed et al. 2019).

## Author Contributions

**Shuai Gong:** conceptualization (supporting), data curation (lead), formal analysis (lead), investigation (lead), writing – original draft (lead), writing – review and editing (equal). **Jia Sun:** data curation (supporting), investigation (supporting), writing – review and editing (equal). **Jianmin Chu:** conceptualization (lead), funding acquisition (lead), project administration (lead), writing – review and editing (equal). **Hongxiao Yang:** conceptualization (supporting), data curation (supporting), investigation (supporting), writing – review and editing (supporting). **Honghao Gan:** conceptualization (supporting), data curation (supporting), investigation (supporting), writing – review and editing (supporting). **Qian Wang:** data curation (supporting), investigation (supporting).

## Ethics Statement

The authors have nothing to report.

## Conflicts of Interest

The authors declare no conflicts of interest.

## Supporting information


**Figure S1:** SNPs density in 1 MB windows along the chromosome of *Tamarix austromongolica* genome.
**Figure S2:** Pairwise comparisons of *F*_ST_ for *Tamarix austromongolica* populations based on whole‐genome data.
**Figure S3:** (A) Elbow method analysis. *K* = 3 represents the point where the rate of decrease in the Within‐Cluster Sum of Squares (WCSS) begins to slow significantly. (B) Distance from each *K* point on the WCSS curve to the line connecting *K* = 1 and *K* = 10 (red line in panel A). The distance is maximal at *K* = 3. (C) Principal component analysis scatter plot for *K* = 3. The three clusters shown are highly consistent with the classification of the three genetic lineages defined in the main text.
**Figure S4:** Genetic diversity and pairwise genetic differentiation indices among three genetic groups of *Tamarix austromongolica*.

**Tables S1:** Sampling information and summary statistics of whole‐genome resequencing data for the 112 T. Austromongolica individuals in this study.
**Table S2:** Summary of SNP datasets used for different population genomic analyses.
**Table S3:** The summaries of gene coordinates for SNP identified in the populations of *T. austromongolica*.
**Table S4:** Optimal model parameters for the dadi simulation of the phylogenetic differentiation of *T. austromongolica*.
**Table S5:** UniProt annotations of 392 CANDIDATE GENES from the Liujiaxia lineage.
**Table S6:** UniProt annotations of 205 candidate genes from the Hetao lineage.
**Table S7:** UniProt annotations of 545 candidate genes from the Sanmenxia lineage.
**Table S8:** GO enrichment analysis of 392 candidate genes from the Liujiaxia lineage.
**Table S9:** GO Enrichment Analysis of 205 Candidate Genes from the Hetao lineage.
**Table S10:** GO Enrichment analysis of 545 candidate genes from the Sanmenxia lineage.

## Data Availability

The raw sequence reads for this study are available in the NCBI Sequence Read Archive (SRA) under BioProject ID (PRJNA1304429). These data are publicly accessible via the link: https://www.ncbi.nlm.nih.gov/bioproject/PRJNA1304429/.
