# Supplementary material for: Whole‐Genome Resequencing Reveals the Demographic History and Adaptive Evolution of Tamarix austromongolica in the Yellow River Basin
Source: Ecol Evol. 2026 Jan 5;16(1):e72666. doi: 10.1002/ece3.72666 (PMC12771681; doi:10.1002/ece3.72666)
Supplement: Supplementary file 1 — Figure S1: SNPs density in 1 MB windows along the chromosome of Tamarix austromongolica genome. Figure S2: Pairwise comparisons of F ST for Tamarix austromongolica populations based on whole‐genome data. Figure S3: (A) Elbow method analysis. K = 3 represents the point where the rate of decrease in the Within‐Cluster Sum of Squares (WCSS) begins to slow significantly. (B) Distance from each K point on the WCSS curve to the line connecting K = 1 and K = 10 (red line in panel A). The distance is maximal at K = 3. (C) Principal component analysis scatter plot for K = 3. The three clusters shown are highly consistent with the classification of the three genetic lineages defined in the main text. Figure S4: Genetic diversity and pairwise genetic differentiation indices among three genetic groups of Tamarix austromongolica. [file ECE3-16-e72666-s001.docx]

**
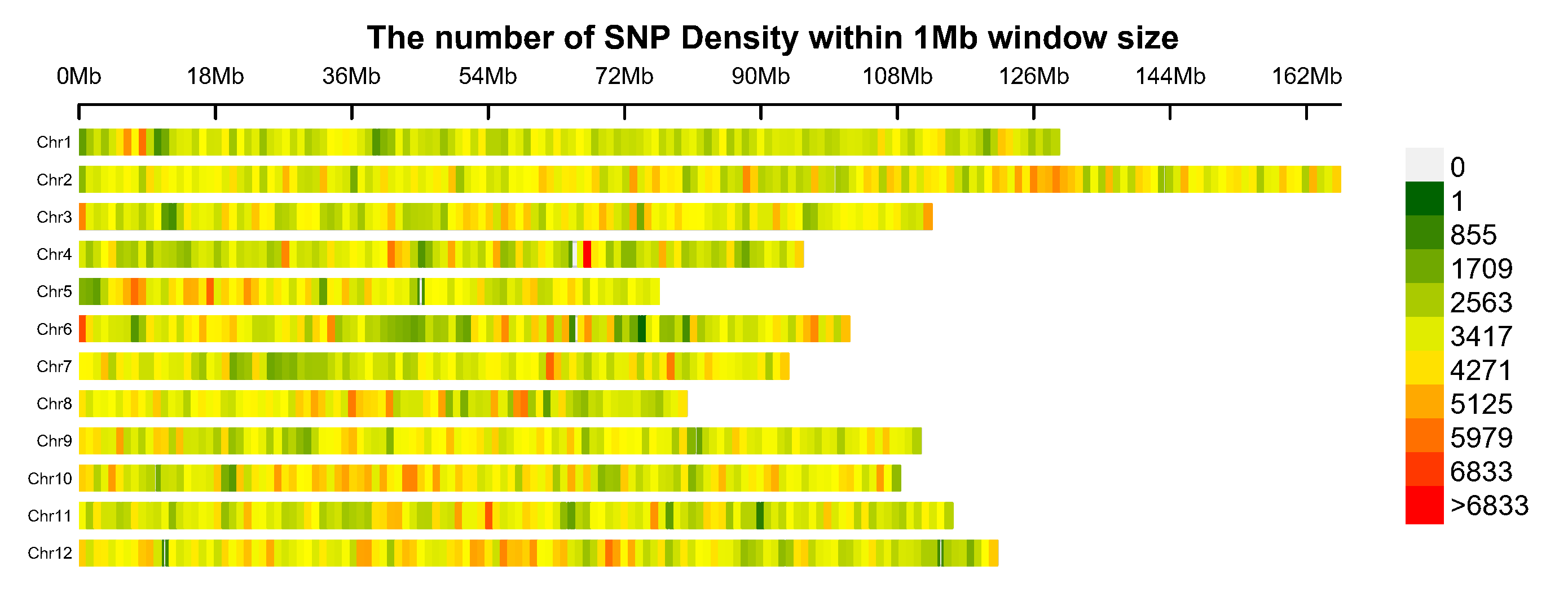
**

**Fig. S1** SNPs density in 1MB windows along the chromosome of *Tamarix austromongolica* genome.

**
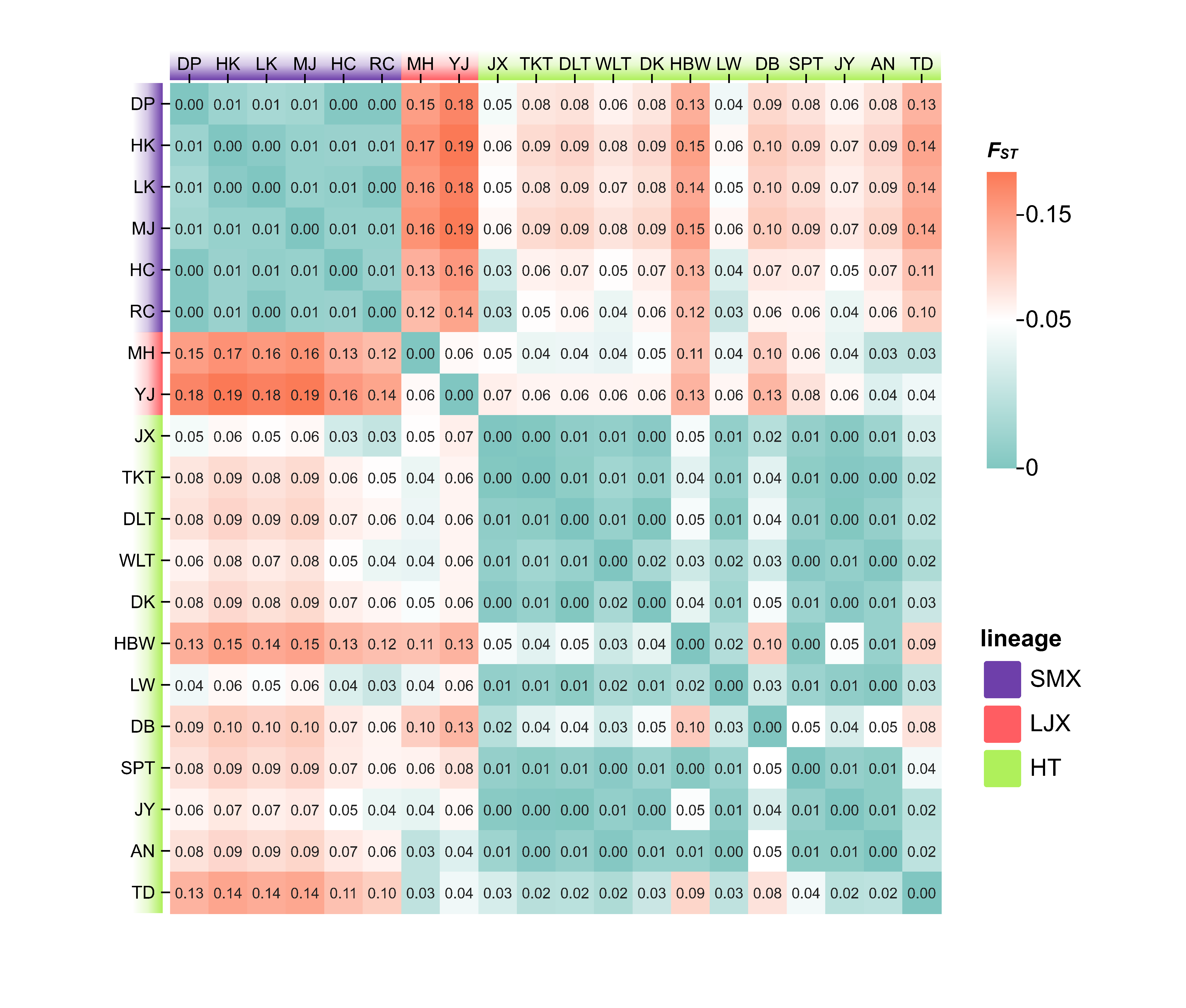
**

**Fig. S2** Pairwise comparisons of *F*st for *Tamarix austromongolica* populations based on whole-genome data.





**Fig. S3** (A) Elbow method analysis. K=3 represents the point where the rate of decrease in the Within-Cluster Sum of Squares (WCSS) begins to slow significantly. (B) Distance from each K point on the WCSS curve to the line connecting K=1 and K=10 (red line in panel A). The distance is maximal at K=3. (C) Principal Component Analysis scatter plot for K=3. The three clusters shown are highly consistent with the classification of the three genetic lineages defined in the main text.

**
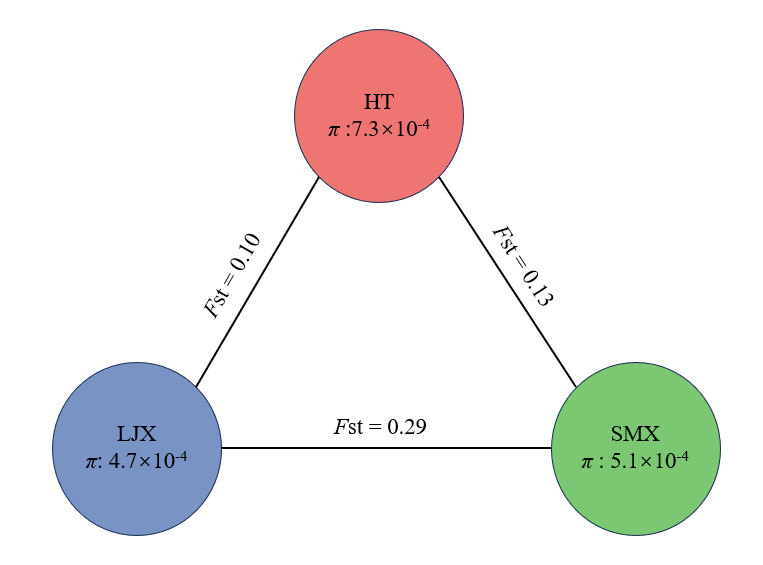
**

**Fig. S4** Genetic diversity and pairwise genetic differentiation indices among three genetic groups of *Tamarix austromongolica.*
